# Complete Resection of a Giant Mediastinal Liposarcoma Extending Into Both Pleural Cavities via Median Sternotomy: A Case Report

**DOI:** 10.1002/ccr3.72017

**Published:** 2026-02-14

**Authors:** Shin‐nosuke Watanabe, Daisuke Kimura, Takahiro Sasaki, Shuta Kimura, Chisaki Ichinohe, Shiori Ono, Hirose Imai, Masahito Minakawa

**Affiliations:** ^1^ Department of Thoracic and Cardiovascular Surgery Hirosaki University, Graduate School of Medicine Hirosaki Japan

**Keywords:** extracorporeal membrane oxygenation, liposarcoma, median sternotomy, mediastinal mass syndrome, mediastinal tumor

## Abstract

Surgical resection is the primary treatment for giant mediastinal liposarcoma. However, aggressive R0 resection involving major nerves can lead to fatal functional loss. Clinicians must balance oncological radicality with functional preservation, as early diagnosis remains the most critical factor for avoiding highly morbid, extensive surgeries.

## 1 Introduction

Mediastinal tumors frequently remain asymptomatic for prolonged periods, leading to delayed detection. Asymptomatic cases are often discovered incidentally during routine chest imaging, whereas symptomatic tumors usually result from compression of adjacent organs. Although certain mediastinal neoplasms, such as malignant lymphomas and germ cell tumors, may respond to chemotherapy or radiotherapy, surgical resection remains the primary treatment. Complete excision is typically required, even for large tumors [1]. We report a case of a giant primary mediastinal liposarcoma occupying most of the mediastinum and both pleural cavities, identified following the onset of dyspnea, and describe its surgical management with a literature review.

## 2 Case History/Examination

**FIGURE 1:**
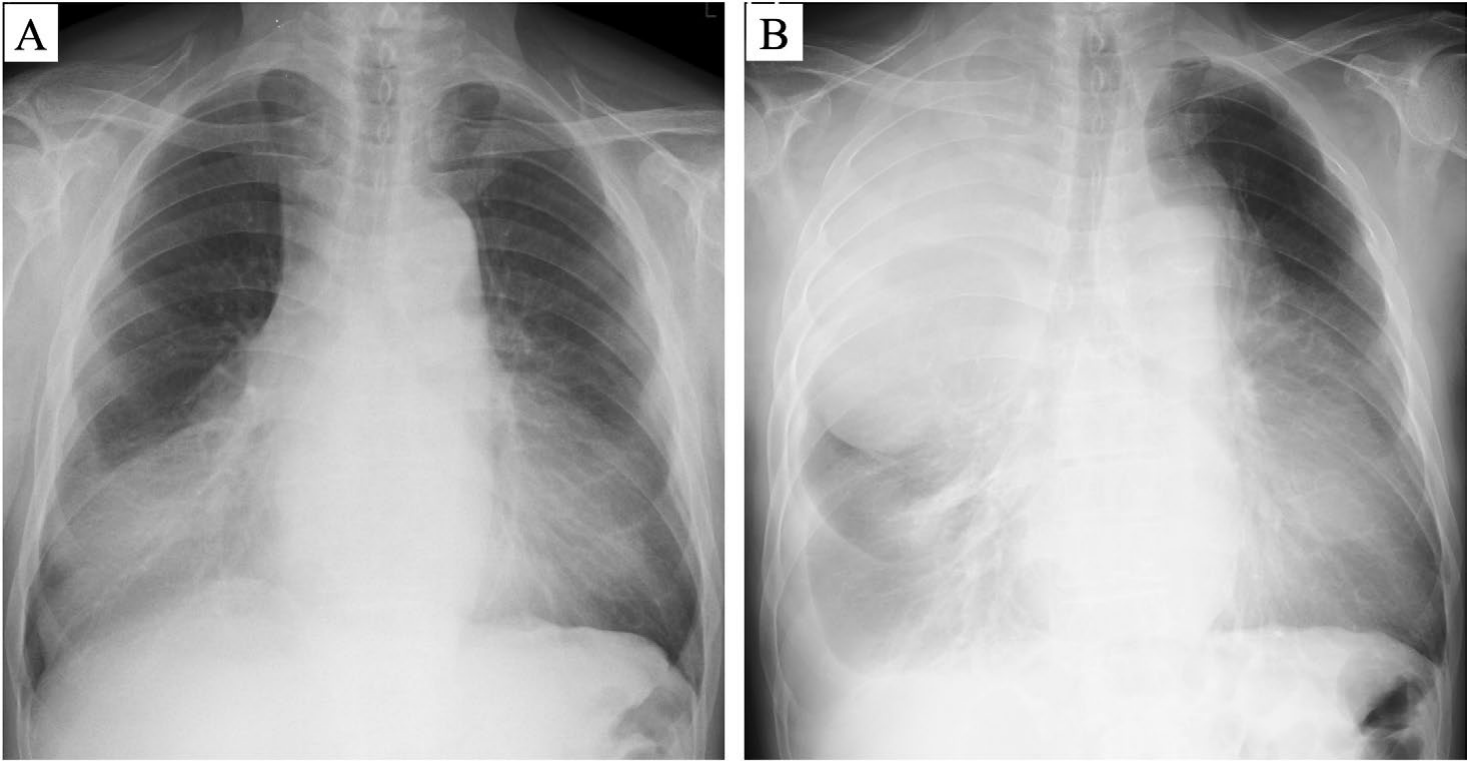
Chest radiograph findings. (A) Chest radiograph obtained three years earlier showing infiltrative shadows in the bilateral lower lung fields. (B) Preoperative chest radiograph demonstrating reduced translucency in both lung fields.

The patient was a 73‐year‐old male with a history of hypertension and atrial fibrillation under regular follow‐up. A chest radiograph obtained three years earlier had revealed an abnormal shadow in the bilateral lower lung fields, but no further evaluation was performed at that time (Figure 1A). He later presented with progressive exertional dyspnea, exacerbated especially in the supine and left lateral decubitus positions. A follow‐up radiograph demonstrated decreased translucency in both lung fields (Figure 1B), prompting referral to our hospital.

## 3 Differential Diagnosis, Investigations and Treatment

To further evaluate the mediastinal mass, multiple imaging modalities were utilized.

**FIGURE 2:**
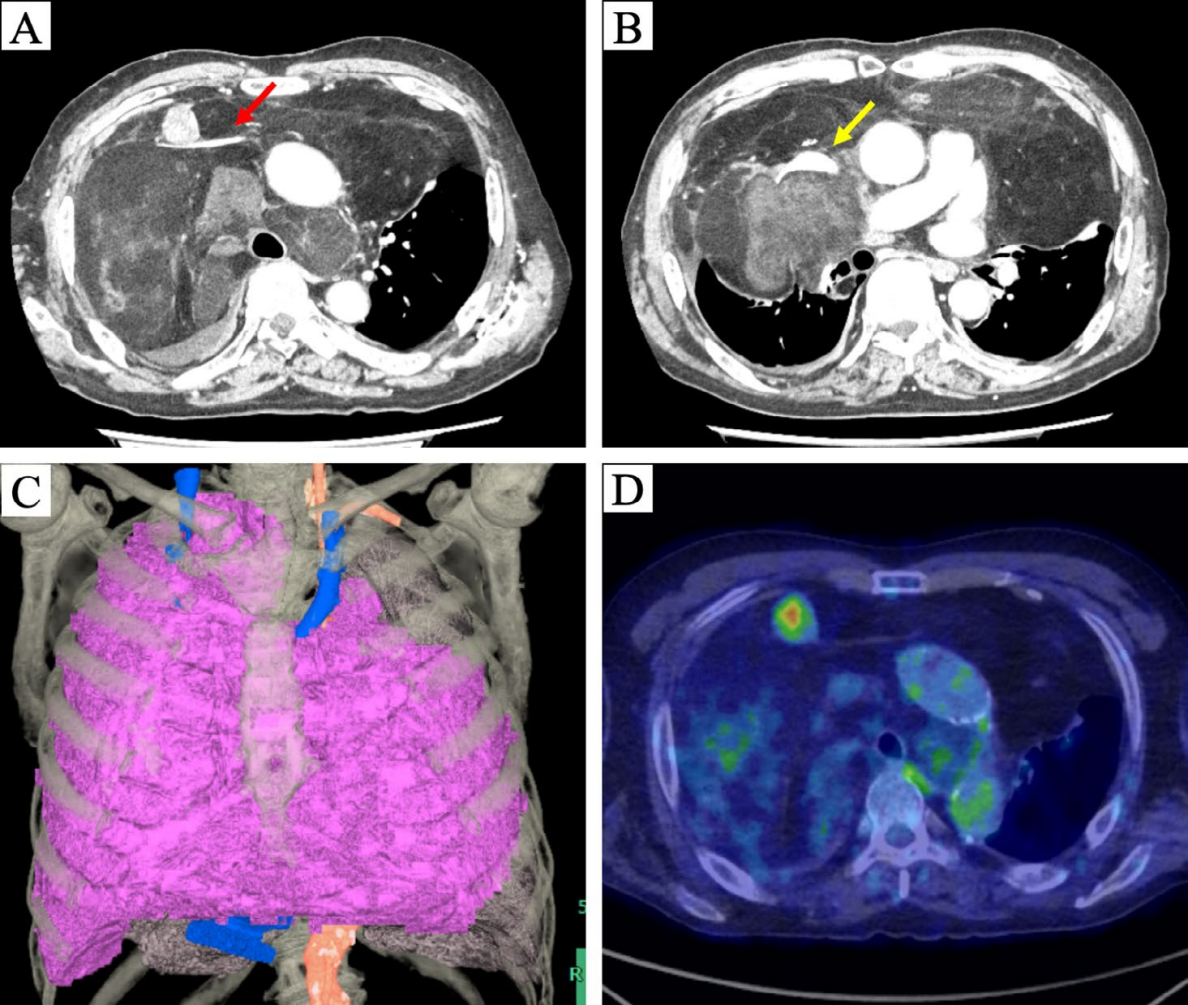
Preoperative imaging findings. (A, B) Chest computed tomography showing a tumor occupying most of the mediastinum and bilateral pleural cavities, encasing major vessels including the left brachiocephalic vein (red arrow) and superior vena cava (yellow arrow). (C) Three‐dimensional reconstructed image demonstrating tumor distribution (purple area). (D) Solid nodule at the confluence of the right and left brachiocephalic veins showing increased fluorine‐18 fluoro‐2‐deoxy‐D‐glucose uptake on positron emission tomography.

Contrast‐enhanced computed tomography revealed a massive 30 × 26 cm tumor occupying both pleural cavities and the anterior–middle mediastinum. The tumor showed infiltrative growth and encased major vessels, including the ascending aorta, right brachiocephalic artery, left common carotid artery, left subclavian artery, right and left brachiocephalic veins, and superior vena cava. Partial atelectasis was present in the left upper lobe, and the right upper and middle lobes were completely collapsed. A 28 × 21 mm solid nodule was identified at the confluence of the right and left brachiocephalic veins (Figure 2A–C). Positron emission tomography–computed tomography (PET‐CT) showed fluorine‐18 fluoro‐2‐deoxy‐D‐glucose uptake at the same site, with a maximum standardized uptake value of 4.7, suggesting possible invasion of the great vessels (Figure 2D). This finding was clinically significant because it raised a strong suspicion of direct invasion into the great vessels, which necessitated careful surgical planning and preparation for potential vascular reconstruction. Although preoperative histologic confirmation was unavailable, imaging findings were highly consistent with liposarcoma. A biopsy was not performed because the patient's severe dyspnea made it difficult to maintain the supine position required for the procedure. Furthermore, given the strong radiological suspicion of liposarcoma and the urgent need for surgical decompression due to the tumor's size, we prioritized early surgical resection over additional diagnostic tests. Since chemotherapy and radiotherapy were considered ineffective for this tumor type, surgical resection was planned.

During induction of general anesthesia, the team anticipated potential circulatory and respiratory failure due to mediastinal mass syndrome (MMS). The patient was positioned semi‐Fowler, and arterial lines were established in both radial arteries. Veno‐arterial extracorporeal membrane oxygenation (ECMO) sheaths were placed in the right femoral vein and left femoral artery for rapid deployment if needed. Muscle relaxants were withheld, and an 8‐mm endotracheal tube was inserted while the patient was awake in the right semi‐lateral decubitus position. No circulatory or respiratory collapse occurred following induction. Surgery commenced with the patient in the supine position.

**FIGURE 3:**
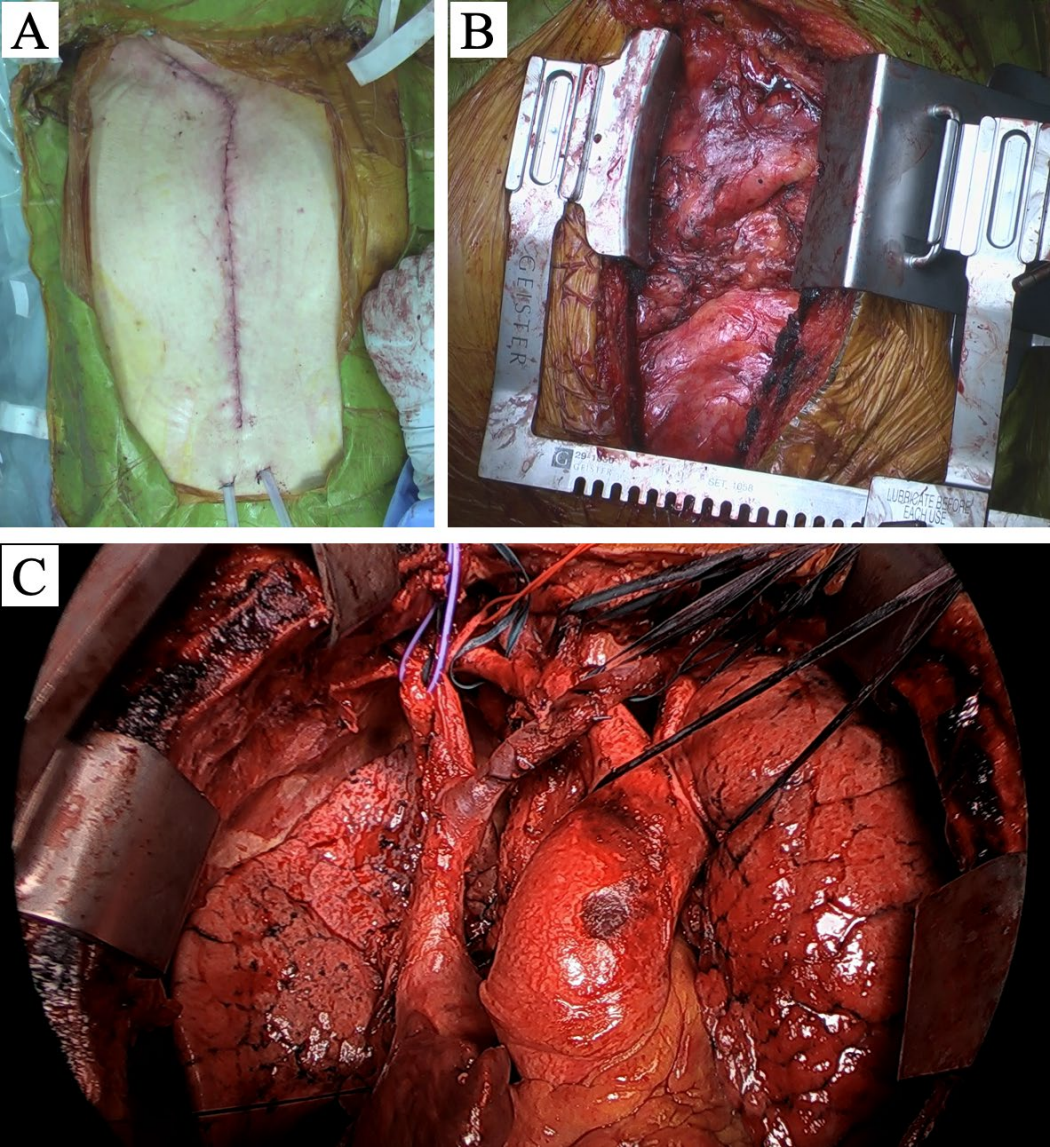
Surgical approach of the present case. (A) Skin incision used during surgery. (B) Intraoperative findings showing tumor extension from the mediastinum into both pleural cavities. (C) The completely resected tumor was divided into six sections.

**FIGURE 4:**
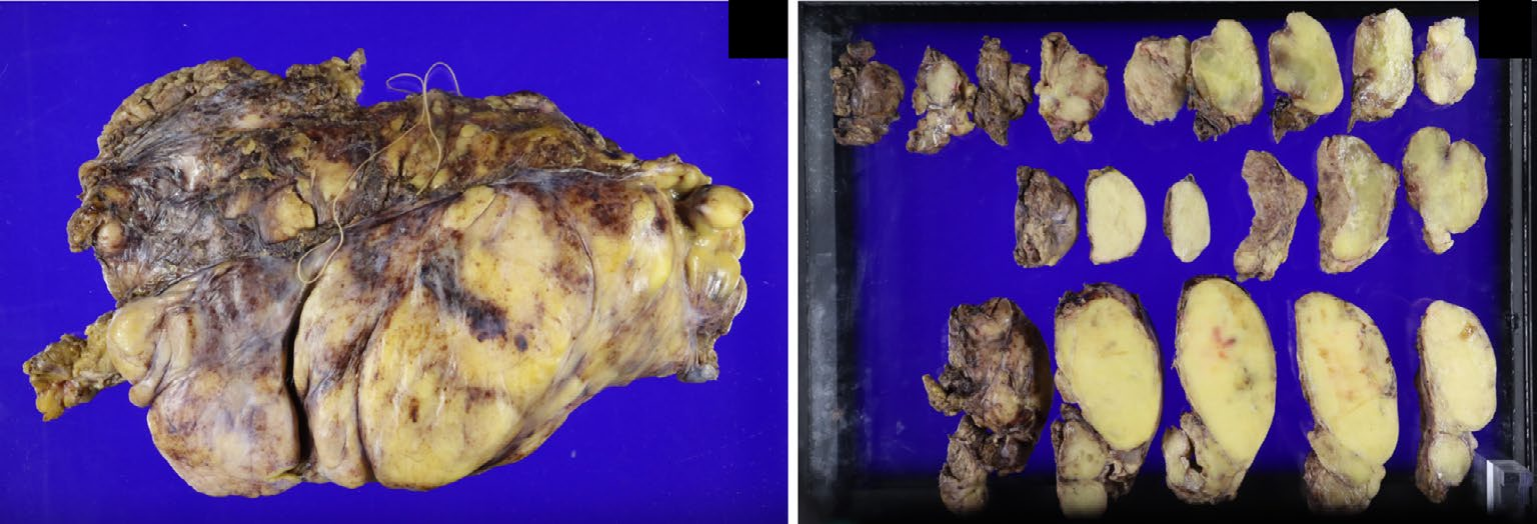
Formalin‐fixed resected tumor divided into six sections with a total weight of 4445 g.

A median sternotomy with right collar incision was performed, and the tumor was dissected bilaterally from the posterior sternal surface to allow sternal retraction. The Speroni One for All Retractor (GEISTER Corporation, Germany) was used for exposure (Figure 3A,B). Serous effusion in the left pleural cavity was collected for cytologic analysis. No macroscopic invasion of either lung was observed. The tumor encased the major vessels as seen on CT, but could be separated without vascular reconstruction. We identified a solid mass at the confluence of the brachiocephalic veins that corresponded to the nodule with high FDG uptake previously identified on PET‐CT (Figure 2D). No macroscopic vascular invasion was detected at this site. The tumor extended cranially to the lateral thyroid gland, caudally to the upper pulmonary artery margins, and posteriorly to the tracheal wall. Complete resection was achieved by dividing the mass into six sections (Figure 3C). The right phrenic nerve was preserved, whereas the left phrenic nerve and both vagus nerves were involved and resected en bloc. The total tumor weight was 4445 g (Figure 4). The operation lasted 12 h 24 min, with intraoperative blood loss of 2809 mL, requiring transfusion of 6 units of red blood cells.

## 4 Conclusion and Results (Outcome and Follow‐Up)

The pathological findings and the subsequent clinical course were as follows.

**FIGURE 5:**
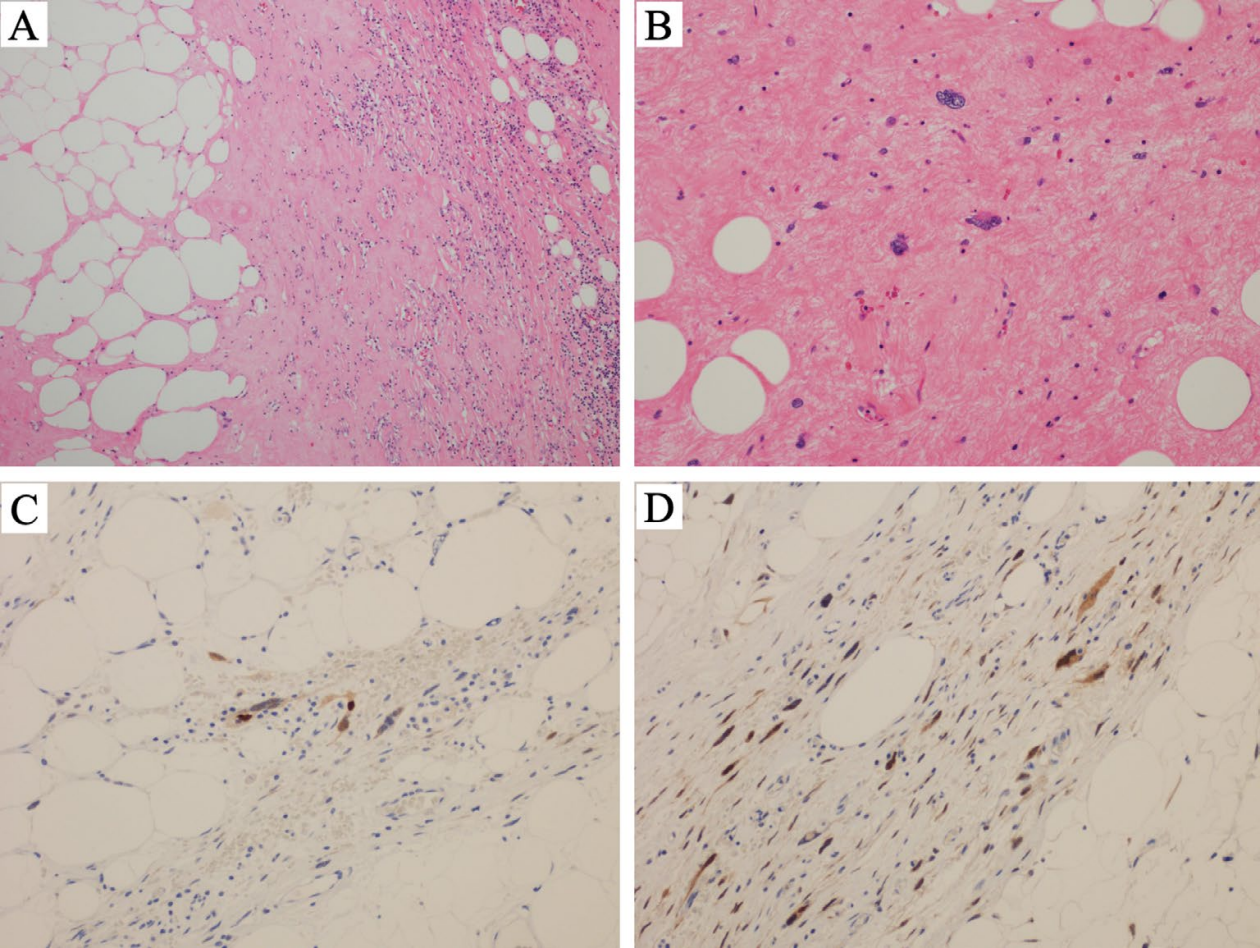
Pathological findings of the resected tumor. Hematoxylin–eosin staining: (A) ×40 magnification showing fibrous septa, variably sized adipocytes, and edematous myxoid stroma. (B) ×200 magnification showing large atypical cells with pleomorphic, hyperchromatic nuclei within fibrous stroma. Immunohistochemical staining: (C) ×200 showing partial tumor cell positivity for MDM2, and (D) ×200 showing positivity for CDK4.

Pathologic examination revealed a large, yellowish‐white mass with fibrous septa and solid nodular components. Microscopically, the tumor consisted of adipocytes of varying sizes separated by fibrous septa and edematous myxoid stroma. Large atypical cells with hyperchromatic, pleomorphic nuclei were present within the stroma (Figure 5A,B). Immunohistochemistry showed partial MDM2 positivity and CDK4 positivity, confirming a diagnosis of well‐differentiated liposarcoma (Figure 5C,D). Cytologic analysis of pleural fluid demonstrated only mesothelial cells, histiocytes, neutrophils, and lymphocytes without malignant cells.

The postoperative course was challenging. Severe hypotension occurred with postural changes, requiring prolonged bed rest. On postoperative day (POD) 7, bilateral pulmonary infiltrates developed. Steroid therapy was initiated for suspected acute respiratory distress syndrome as the primary diagnosis. Additionally, broad‐spectrum antibiotics were administered because the possibility of concurrent bacterial pneumonia could not be ruled out. However, mechanical ventilation could not be discontinued. A tracheostomy was performed on POD 23, and enteral nutrition via nasogastric tube was started along with total parenteral nutrition. Gastric dysmotility led to pyloric obstruction, necessitating endoscopic placement of a double‐elementary diet tube. Enteral feeding resumed, but frequent diarrhea prevented adequate nutritional recovery, and serum albumin remained low. Persistent peripheral edema and circulatory instability required catecholamine support until POD 37. Pleural effusion and urinary tract infections were managed with drainage and antibiotics. Ultimately, the patient died of septic shock secondary to acute pneumonia on POD 94.

We presented a case of complete resection of a giant liposarcoma extending into both thoracic cavities through only a median sternotomy. Given the complex postoperative course, the extent of resection should be carefully balanced against potential functional compromise.

## 5 Discussion

Liposarcoma originates from primitive mesenchymal cells and most frequently develops in the extremities or retroperitoneum. Mediastinal cases are exceedingly rare, representing < 1% of all liposarcomas [2]. Histologically, liposarcoma is classified into four subtypes: well‐differentiated, myxoid, pleomorphic, and dedifferentiated. Well‐differentiated liposarcoma typically exhibits slow growth, whereas pleomorphic and dedifferentiated types demonstrate more aggressive behavior and higher metastatic potential [2, 3]. Generally, well‐differentiated liposarcomas expand with compressive margins. However, tumors arising in the retroperitoneum, mediastinum, or head and neck, as well as sclerosing variants or those containing dedifferentiated components, may invade adjacent structures [3]. Therefore, early diagnosis and intervention are essential to optimize patient outcomes. Delayed evaluation often allows the tumor to reach a massive scale, leading to the inevitable infiltration of vital structures such as major nerves and vessels. In such cases, the radical resection required for oncological cure may cause severe functional compromise and postoperative complications. Consequently, prompt management of even asymptomatic mediastinal shadows is critical to avoid the need for aggressive, functionally devastating surgery. The necessity for early surgical intervention is further underscored by the distinct treatment resistance of liposarcomas compared to other mediastinal neoplasms. Unlike thymic carcinomas, lymphomas, or other mediastinal malignancies that may respond to multimodal treatment, liposarcomas, particularly the well‐differentiated subtype, are generally resistant to chemotherapy and radiotherapy [1, 2, 3]. Therefore, the concept of neoadjuvant therapy is rarely applicable, and surgical resection remains the primary treatment. Even when the tumor involves central structures like the aorta, vena cava, or trachea, surgical resection is still considered the treatment of choice rather than palliative systemic therapy because the disease is typically local rather than systemic. Achieving a complete (R0) resection is the most critical factor for improving survival and can be curative, yielding low recurrence rates and favorable long‐term outcomes [4, 5].

Mediastinal liposarcomas often enlarge silently over several years. By the time symptoms such as chest pain, dyspnea, or cough appear, the tumors are typically massive [6, 7], necessitating meticulous perioperative management and surgical planning. During anesthesia induction, careful attention must be directed toward MMS, in which giant anterior or superior mediastinal tumors cause acute circulatory collapse or respiratory failure due to mechanical compression triggered by sedatives, muscle relaxants, or positional changes [8, 9]. Preoperative assessment should determine a safe induction position by evaluating symptom provocation and vital sign fluctuations. CT is essential for assessing airway narrowing, bronchial diameter, and endotracheal tube selection [8, 10]. Preparation for rapid ECMO initiation is critical for emergencies such as difficult intubation or sudden MMS onset [8, 11]. In the present case, the patient experienced dyspnea in the supine and left lateral decubitus positions but remained stable in the right lateral position. Therefore, anesthesia was induced in the right semi‐lateral position using an 8‐mm tube without muscle relaxants. The patient was subsequently placed supine without circulatory collapse or respiratory failure. Although ECMO was not required, preparation was indispensable for procedural safety.

For giant anterior mediastinal tumors, securing adequate exposure of the pulmonary hilum and thoracic cavity is essential. When tumors extend into both pleural cavities or invade the lungs, hemi‐clamshell or clamshell approaches may be effective [12, 13, 14]. The clamshell approach—bilateral anterolateral thoracotomy combined with transverse sternotomy—provides simultaneous access to both pleural cavities and the anterior mediastinum. It is therefore suitable for resection of extensive mediastinal tumors, bilateral pulmonary disease, and lung transplantation. However, disadvantages include significant invasiveness, severe postoperative pain, and impaired respiratory function, as well as risks of bleeding, infection, chest wall instability, and cosmetic issues. Median sternotomy remains the standard approach for anterior mediastinal tumors. In this case, combining thoracoscopy with the Speroni One for All Retractor—an instrument designed for sternal spreading, internal thoracic artery harvesting, and direct cardiac access—provided excellent visualization of both pleural cavities [15]. This device proved particularly advantageous. Although extension to a transverse incision was considered depending on tumor spread, complete resection was achieved through a single median sternotomy. However, an en bloc resection was technically impossible because of the massive size of the tumor and its extensive encasement of the great vessels. The tumor was therefore divided into six sections during removal to maintain a clear surgical field and prevent injury to vital structures. While intraoperative fragmentation carries a risk of tumor dissemination, this approach was necessary to prioritize the safety of the vascular dissection. Furthermore, the left phrenic and both vagus nerves were found to be macroscopically encased by the tumor. We prioritized an R0 resection and decided to sacrifice these nerves without performing intraoperative frozen sections. In retrospect, this decision remains a point of critical reflection.

Postoperative management was complicated by the consequences of this extensive resection of perivascular connective tissue and both vagus nerves, which contributed to hemodynamic instability. Within the unified thoracic cavity, unsupported great vessels appeared compressed by the lung or sternum during postural changes, causing stenosis, occlusion, and profound hypotension. Prolonged bed rest was required, and the loss of the vagus nerve abolished the cough reflex, impairing sputum clearance [16, 17] and necessitating daily bronchoscopic suctioning. Severe gastrointestinal dysmotility further worsened nutrition and muscle strength [18]. Reduced colloid osmotic pressure, combined with thoracic duct and lymphatic vessel resection disrupting lymphatic drainage, led to persistent edema and pleural effusion. This case highlights a critical clinical lesson. Although complete tumor excision remains the gold standard for a potential cure, the extent of resection must be carefully weighed against the risk of devastating functional loss. Especially when sacrificing major nerves, intraoperative frozen sections should be utilized to confirm the necessity of the resection. Ultimately, functional preservation may sometimes be the more prudent choice for the patient's overall survival.

## Author Contributions

All authors participated in the surgery and perioperative care. S.‐n.W. was the primary contributor to manuscript drafting. D.K. and M.M. critically revised the manuscript. All authors reviewed and approved the final version of the manuscript.

## Funding

The authors have nothing to report.

## Disclosure

Permission to Reproduce Material: All figures and images are original and have not been previously published.

## Ethics Statement

The authors have nothing to report.

## Consent

Written informed consent for publication of this case report and accompanying images was obtained from the patient's family.

## Conflicts of Interest

The authors declare no conflicts of interest.

## Data Availability

Data sharing is not applicable, as no datasets were generated or analyzed during this study.
